# Characterizing cancer cells with cancer stem cell-like features in 293T human embryonic kidney cells

**DOI:** 10.1186/1476-4598-9-180

**Published:** 2010-07-08

**Authors:** Bisrat G Debeb, Xiaomei Zhang, Savitri Krishnamurthy, Hui Gao, Evan Cohen, Li Li, Angel A Rodriguez, Melissa D Landis, Anthony Lucci, Naoto T Ueno, Fredika Robertson, Wei Xu, Lara Lacerda, Thomas A Buchholz, Massimo Cristofanilli, James M Reuben, Michael T Lewis, Wendy A Woodward

**Affiliations:** 1 Department of Radiation Oncology, The University of Texas MD Anderson Cancer Center, Houston, TX, USA; 2 Lester and Sue Smith Breast Center and Department of Molecular and Cellular Biology, Baylor College of Medicine, Houston, TX, USA; 3 Department of Pathology, The University of Texas MD Anderson Cancer Center, Houston, TX, USA; 4 Department of Hematopathology, The University of Texas MD Anderson Cancer Center, Houston, TX, USA; 5 Department of Surgical Oncology, The University of Texas MD Anderson Cancer Center, Houston, TX, USA; 6 Department of Breast Medical Oncology, The University of Texas MD Anderson Cancer Center, Houston, TX, USA; 7 Department of Experimental Therapeutics, The University of Texas MD Anderson Cancer Center, Houston, TX, USA

## Abstract

**Background:**

Since the first suggestion of prospectively identifiable cancer stem cells in solid tumors, efforts have been made to characterize reported cancer stem cell surrogates in existing cancer cell lines, and cell lines rich with these surrogates have been used to screen for cancer stem cell targeted agents. Although 293T cells were derived from human embryonic kidney, transplantation of these cells into the mammary fat pad yields aggressive tumors that self-renew as evidenced by serial xenograft passages through transplantation. Herein we fully characterize cancer stem cell-like features in 293T human embryonic kidney cells.

**Results:**

293T cells can be readily cultured and passaged as spheres in serum-free stem cell promoting culture conditions. Cells cultured in vitro as three-dimensional spheres (3D) were shown to contain higher ALDH1 and CD44+/CD24- population compared to monolayer cells. These cells were also resistant to radiation and upregulate stem cell survival signaling including β-catenin, Notch1 and Survivin in response to radiation. Moreover, 3D spheres generated from the 293T cells have increased expression of mesenchymal genes including vimentin, n-cadherin, zeb1, snail and slug as well as pro-metastatic genes RhoC, Tenascin C and MTA1. In addition, microRNAs implicated in self-renewal and metastases were markedly reduced in 3D spheres.

**Conclusions:**

293T cells exhibit a cancer stem cell-like phenotype when cultured as 3D spheres and represent an important research tool for studying the molecular and biological mechanisms of cancer stem cells and for testing and developing novel targets for cancer therapy.

## Background

The cancer stem cell theory proposes that a subpopulation of cells, the cancer stem cells, exist in solid tumors as well as cancers of hematopoietic origin and constitute a reservoir of self-sustaining cells with the exclusive ability to self-renew and maintain the tumor. These cancer stem cells have the capacity to both divide and expand the cancer stem cell pool and to differentiate into the heterogeneous non-tumorigenic cancer cell types that constitute the bulk of the cancer cells within the tumor [1]. Cancer stem cells have been proposed to play a role in tumorigenesis and metastasis [2-4] as well as in resistance to radiation and chemotherapy [5-9]. Thus, it may be necessary to target and eliminate these cells to eradicate cancers.

Cancer stem cells have been identified in a mounting number of human malignancies. Using approaches developed in the hematopoietic malignancies, Clarke and colleagues demonstrated the existence of a subpopulation of tumorigenic cells (or tumor-initiating cells), isolated from breast cancer pleural effusions by limiting dilution transplantation of CD44+/CD24-/lineage- cells into the mammary fat pad of immunocompromised mice [10]..Importantly, Dontu et al. adapted a sphere culture system described in the central nervous system [11] by growing primary breast epithelial cells in serum-free medium containing EGF and FGF2 in suspension culture and demonstrated enrichment of the tumor-initiating population. This enriched population is suitable to demonstrate in vitro self-renewal and multi-lineage differentiation potential [12]. This assay has been adapted to study cancer stem/progenitor cells in breast cancer cell lines as well as in colon, brain and pancreatic cancer tissues [11,13-18]. Another marker of the cancer stem cell population was recently established by Ginestier et al. who showed that aldehyde dehydrogenase 1 (ALDH1)-positive cells from human breast tumors were the tumorigenic cancer-initiating population [19]. High ALDH activity has also been used to define cancer stem cell populations in other solid tumors including colon, liver and pancreatic cancers [20-22]. Numerous studies suggest that cell lines can be useful in vitro tools to study these populations, and at this time the percentage of cancer stem cell markers has been characterized in numerous cancer cell lines [15,17]. These efforts have led to the use of appropriately selected cell lines rich in the surrogate phenotypes to screen for agents that target these cancer stem cell surrogates [23]; however the number of lines expressing each of the surrogates reported thus far is few.

We observed several key biologic features of purported cancer stem cells in human embryonic kidney cells 293T (also designated HEK 293T), a widely used cell line derived through the sheared Adenovirus 5 (Ad5) DNA transformation [24]. Most importantly, tumors from these cells self-renew as evidenced by serial xenograft passage through transplantation into the cleared mammary fat pad continued over eight generations. Guan et al previously showed that primary HEK 239T cells form tumors that could be inhibited by RbAP46 [25], and Shen et al. demonstrated that tumor forming efficiency had increased with increasing passage of these cells [26], but serial self-renewal has not been reported. We hypothesized that these embryonic, tumorigenic cells may be useful to study cancer stem cells and add to the arsenal of cell lines with significant cancer stem cell surrogate representation to facilitate the study of targeted agents against this population. Here we focused on the cancer stem cell surrogates described in breast tumor models and transplanted tumor cells into the cleared mammary fatpads to examine in vivo tumor pathology. We report that 293T cell readily form spheres that are enriched with cancer stem cell surrogates ALDH1 and CD44+/CD24- cells and express proteins associated with the epithelial-mesenchymal transition and stem cell developmental pathways. They are also depleted in the microRNA that has been implicated in cancer stem cell self-renewal. We propose that this is a valuable cell line to study cancer stem cell surrogates identified in human cancers as well as the plasticity of transformed embryonic cells.

## Results

### 293T cells form spheres in stem cell promoting culture that are enriched in cancer stem cell markers

**Figure 1:**
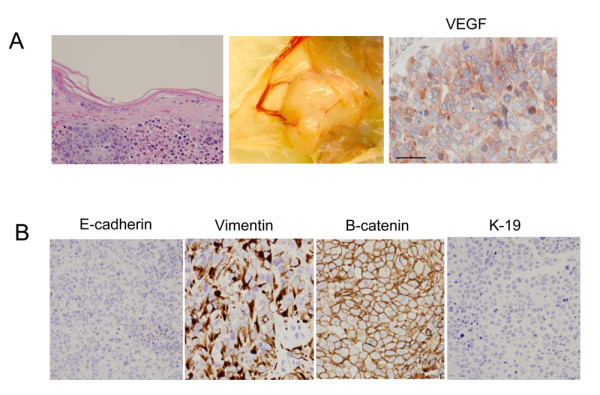
**Transplantation of 293T cells into the cleared mammary fat pad of immunocompromised mice yields serially transplanting tumors**. A) Skin overlying grossly involved tumor is necrotic and abnormal and tumors demonstrate gross tumor vasculature and are diffusely positive for vascular endothelial growth factor (VEGF). B) By immunohistochemical analysis tumor xenografts are strongly vimentin positive, E-cadherin negative and beta-catenin positive at the membrane while K19, an epithelial marker is completely negative.

When 293T cells were transplanted into the cleared fat pads of 3 week old SCID/Beige mice, all (10/10) glands developed tumors at 14 days. These tumors were serially passaged through six transplant generations and are phenotypically stable. Hemotoxylin and eosin staining was performed and examined by a clinical breast pathologist. Histology was indistinguishable from high grade adenocarcinoma of the breast (although the histological appearance of high grade poorly differentiated tumor is not specific only to breast tumors). All tumors had evident tumor vasculature and expressed VEGF (Figure 1A). Necropsy was performed on all mice at the time of sacrifice and no metastatic disease was identified in solid organs. Bone marrow aspirates from generation two mice were negative for malignant cells. Tumors were negative for the epithelial markers e-cadherin and cytokeratin 19 while they were positive for vimentin and β-catenin (Figure 1B), features associated with poorly differentiated tumors.

**Figure 2:**
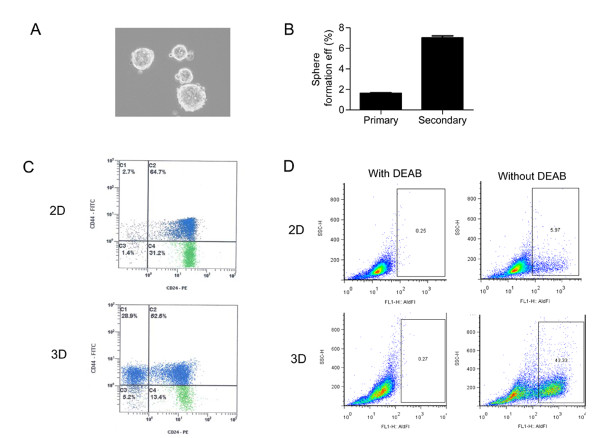
**293T cells form spheres in suspension culture that exhibit cancer stem cell phenotype**. A, B) Sphere formation in 293T cells seeded in self renewal promoting suspension culture conditions. Passage of 293T spheres gives rise to higher proportion of secondary spheres. C) Flow cytometric analysis of expression of CD24 and CD44 in 293T cells. Cells were cultured under adherent (2D) or tumor-initiating cells enrichment condition (3D). There is an increase of CD44+/CD24- population in cells cultured in 3D vs. 2D culture conditions. D) Flow cytometric analysis of ALDH activity showed that the 3D culture condition enriches for ALDH-positive cells compared to the 2D culture of 293T cells.

Given the in vivo observation that 293T tumors serially transplant and self-renew, we characterized the cell line for its cancer stem cell properties. 293T cells seeded in the self-renewal promoting suspension culture conditions formed round spheres of varying sizes and these spheres can be passaged to give a higher proportion of secondary spheres (Figure 2A, B). To characterize further the stem cell component of the spheres, flow cytometry was used to measure cells bearing the tumor-initiating phenotype CD44+/CD24- and ALDH activity. As shown in Figures 2C and 2D, CD44+/CD24- cells represent an average of 4% ± 2% cells in 2D culture and 22% ± 10% cells in 3D culture (5-fold increase, P = 0.003) and 3D spheres showed a 7-fold ALDH activity compared to 2D cells (6% vs. 43%). Further, tumors from 293T were dissociated into single cells and assessed for cancer stem cell surrogates using fresh cells or cells propagated under sphere forming conditions. CD44+/CD24- cells comprised an average of 0.25% (N = 2) from primary tumors and 16.4% (N = 4) from tumor derived sphere culture, a 65-fold increase. ALDH increased comparably from 0.2% in the primary tumor to an average 17.5%. Individual tumor data are summarized in table 1.

**Table 1:** Characterizing breast cancer stem cell surrogates in spheres derived from 293T tumors

Tumor ID	CD44+/CD24-	CD44-/CD24+	CD44+/CD24+	CD44-/CD24-	ALDH+/-DEAB
293T	0.4	0.2	0	98.6	0.2
293T	0.1	37.4	0	52.6	0.2
293T Sphere -1	5.5	2.3	0	92.4	24.6
293T Sphere -2	17.4	1	0	80.2	27.7
293T Sphere -3	24.2	0.2	0	72.1	12.1
293T Sphere -4	18.3	3.2	0.1	78.5	5.6

**Figure 3:**
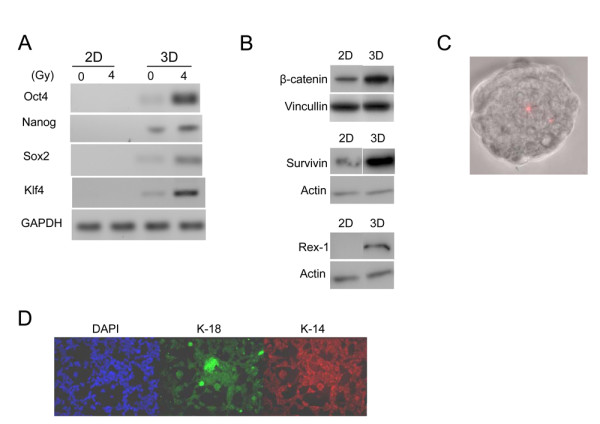
**Spheres from 293T cells express embryonic transcription factors implicated in self-renewal**. A) RT-PCR demonstrated higher expression of embryonic stem cell specific genes including Oct4, Nanog, Sox2 and Klf4 in 3D spheres of 293T cells but no expression was observed in 2D monolayer cells. B) Genes implicated in promoting survival of cancer stem cells such as β-catenin, survivin and Rex-1 were upregulated in 3D spheres vs. 2D monolayer cells. C) Serial passage for more than 4 weeks without additional PKH-26 label (Red) reveals serial dilution to a single long term label-retaining cell per sphere suggestive of asymmetric self-renewal. D) Immunocytochemistry reveals the majority of 293T cells as keratin 14 positive.

Using RT-PCR to examine the expression of embryonic stem cell gene mRNAs in cells derived from spheres (3D) and compare them to cells maintained as adherent monolayer culture we found 3D spheres selectively expressed embryonic stem cell transcription factors Oct4, Nanog, Sox2 and Klf4 (Figure 3A) genes whose expression was associated with reprogramming of adult fibroblasts into induced pluripotent stem cells [27-29]. We also examined stem cell signaling and downstream effectors in 2D versus 3D culture as a surrogate comparison of differentiated versus progenitor enriched culture. β-catenin, survivin and Rex-1 were upregulated in 3D culture (Figure 3B) in protein lysates obtained from 293T cells cultured as spheres vs. monolayer cells. Rex-1 is a target gene of the stemness marker Oct4 and thus its expression in the 3D spheres could be a functional readout of Oct4 activity. Moreover, the self-renewal feature of the 293T spheres was assessed using the label retaining fluorescent membrane dye, PKH-26. Self-renewal in sphere culture is expected to generate both heterogeneous spheres containing self-renewing cells and committed lineage specific spheres derived from self-renewing cells. In spheres from 293T cells using PKH-26 label retention as a marker for quiescent cells, spheres were identified after 4 passages that retain a single label-retaining cell (Figure 3C). Furthermore, immunostaining of 293T cells showed that the majority of cells stain with the basal cytokeratin K14 while few cells stain for the luminal marker K18 (Figure 3D) suggesting that 293T cells have early differentiating cell characteristics.

### Spheres generated from 293T cells are resistant to therapy

**Figure 4:**
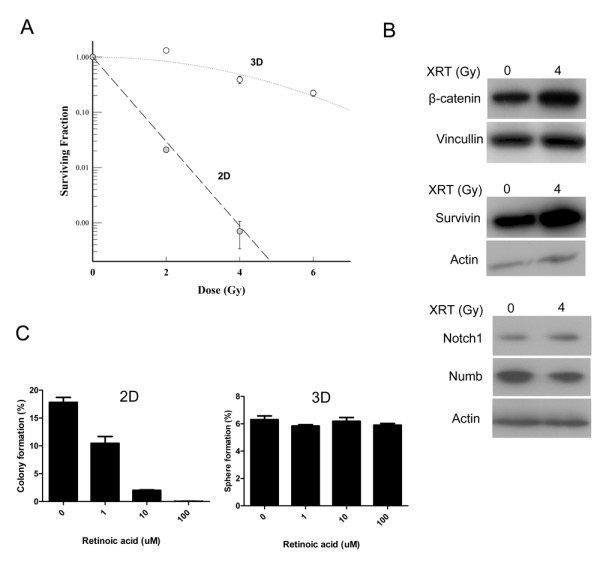
**Spheres generated from 293T cells are resistant to therapy**. A) Clonogenic/mammosphere formation assay of 293T cells cultured under 2D or 3D promoting culture conditions. Cells in 3D are more resistant to radiation than cells grown in 2D. Cells were grown in 2D or 3D culture conditions and irradiated with single, increasing doses (0, 2, 4, 6 Gy) of radiation. B) Upregulation of genes implicated in radioresistance including β-catenin, Notch and Survivin in 3D cells 48 hours after 4 Gy of irradiation. C) 3D cultured cells are also more resistant to chemotherapeutic drugs like retinoic acid compared to 2D. Similar results were obtained with Taxol (data not shown). Indicated concentrations of drugs were added to the media until the number of colonies or spheres were counted.

Following the observation that 293T cells from the sphere culture are enriched in cancer stem cell markers, we examined differences in resistance to radiation- or chemo-therapy between 2D and 3D cultures. Using the 3D mammosphere/2D clonogenic assay method that has been recently demonstrated by our lab [16], we found that cells in 3D culture are significantly more resistant to radiation than the same cells grown in 2D culture (Figure 4A) and second generation spheres are more resistant than primary spheres (data not shown). Moreover, β-catenin, Notch and survivin were all upregulated in 3D culture cells 48 hours after 4 Gy of irradiation compared to the unirradiated cells while Numb, a negative regulator of Notch, is downregulated with radiation (Figure 4B). Similarly Retinoic acid, a compound that has been used as a cancer chemotherapeutic agent, significantly reduced the number of colonies in monolayer culture but did not decrease sphere formation (Figure 4C) indicating that the 3D cells can be resistant to chemotherapeutic agents.

### Epithelial-Mesenchymal Transition (EMT) and pro-metastatic genes are upregulated in 3D spheres of 293T cells

**Figure 5:**
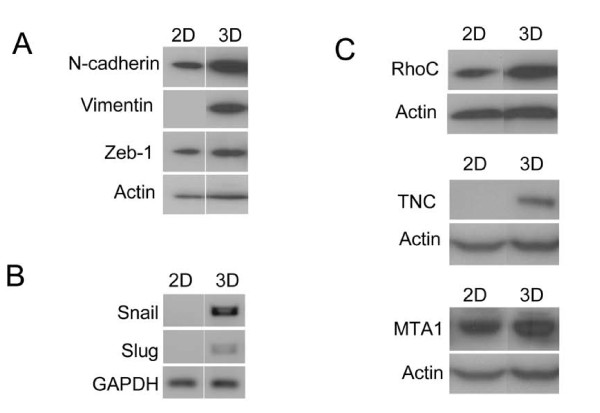
**EMT and pro-metastatic genes are upregulated in 3D spheres of 293T cells**. A) 293T spheres show upregulation of markers of the mesenchymal phenotype in the epithelial-mesenchymal transition such as higher n-cadherin, vimentin, and zeb-1 and low E-cadherin compared to 2D cells. B) RT-PCR demonstrated higher expression of EMT promoting genes Snail and Slug in 3D sphere culture vs. 2D monolayer culture. C) Western blot demonstrated upregulation of known proteins important in promoting metastasis such as RhoC, Tenascin-C and MTA1 in 3D spheres compared to 2D culture.

In view of the recent findings that EMT increases the stem cell population in normal and malignant mammary epithelial cells as well as in pancreatic and colorectal cancer cells [13,18], we investigated EMT gene expression patterns by western blot in the 2D versus 3D culture as a surrogate comparison of differentiated versus stem/progenitor enriched culture. Figure 5A shows that the mesenchymal gene markers n-cadherin, vimentin and zeb1 were upregulated in 293T cells cultured in serum-free sphere promoting conditions vs. the standard 2D culture conditions. Snail and slug, other EMT genes, were also upregulated in the 3D culture compared to the 2D with RT-PCR (Figure 5B).

We also assessed differences in 2D vs. 3D for the expression pattern of genes implicated in promoting invasion, migration and metastasis of cancer cells. RhoC is upregulated in 3D spheres of 293T cells as compared to monolayer cells (Figure 5C). RhoC has been identified as an important player in metastasis [30,31] and its expression correlates with metastatic spread of various types of carcinomas [32,33]. Similarly, Tenascin C and MTA1 which are also associated with invasion and migration of cancer cells [34-36] were upregulated in 3D vs. 2D 293T cells (Figure 5C).

### MicroRNAs implicated in self renewal and metastasis are markedly reduced in 3D spheres

**Figure 6:**
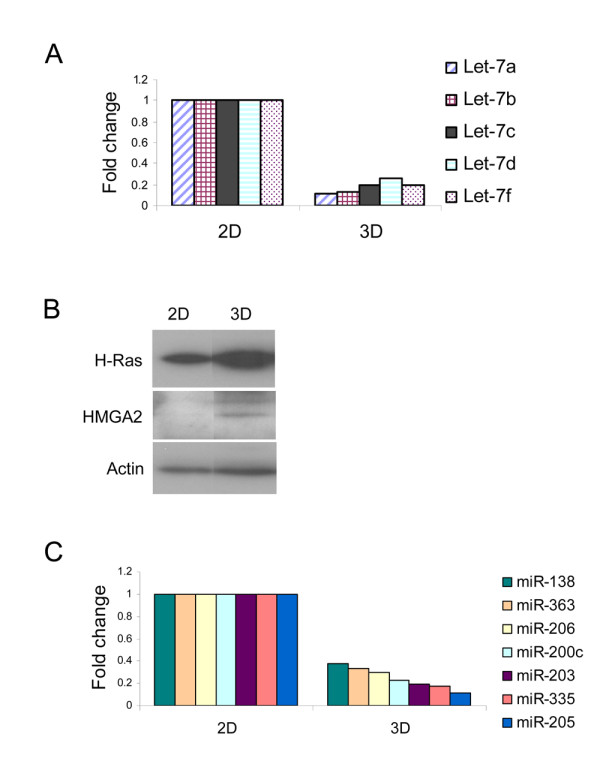
**MicroRNAs implicated in self-renewal and metastases are markedly reduced in 3D spheres**. A) Higher expression of relative Let-7 microRNA levels was seen in 2D vs. 3D sphere culture. RNA was extracted from 293T cells cultured in 2D and 3D conditions and subjected to real time PCR using miRNA primers from Ambion. B) H-Ras and HMGA2, two known targets of Let-7 microRNAs, were expressed higher in 3D than 2D cells by western blot. C) MicroRNAs known to regulate EMT are expressed at very low levels in 3D culture. RNA was extracted from 293T cells cultured in 2D and 3D conditions and subjected to quantitative PCRarray from SABiosciences.

Following the findings that 3D spheres from 293T cells exhibit the cancer stem cell phenotype as well as express EMT and pro-metastatic genes, we examined for differences in the expression of microRNAs that are associated with self-renewal and metastases. It has been demonstrated that the let-7 microRNA family regulates the self-renewal of breast cancer stem cells [37]. In line with these findings, we found that Let-7 microRNAs are highly reduced in 3D stem cell promoting cultures in 293T cells compared to the 2D monolayer cells (Figure 6A) while two known Let-7 target genes, H-Ras and HMGA2, were upregulated in 3D vs. 2D (Figure 6B). Likewise, the microRNAs miR-200c, miR-205, miR-206 and miR-335 previously reported to be involved in invasion and metastasis [38-40] are reduced in the 3D stem cell promoting cultures in 293T cells. Moreover, miR-138, miR-203 and miR-363 previously uncharacterized in metastases are also downregulated in 3D vs. 2D cells (Figure 6C). Table 2 summarizes the miRNAs downregulated in 3D culture of 293T cells and the validation of predicted genes by western blot or RT-PCR.

**Table 2:** MicroRNAs downregulated in 3D culture and their predicted target genes

miRNAs	Predicted target genes (related to invasion and metastasis)	Targets validated in 3D culture
miR-138	Vimentin	Yes
	RhoC	Yes
	Sox-4	No
	Zeb2	No

miR-363	Snail	Yes
	Zeb-2	No

miR-206	Slug	Yes
miR-200c	Zeb-1	Yes

miR-203	Slug	Yes
	TGFb3	No

miR-205	Zeb-2	No

miR-335	TNC	Yes
	Sox-4	No

## Discussion

We propose 293T is a valuable tool to study cancer stem/progenitor features in that it rapidly generates both spheres and tumors that are readily serially passaged, and maintains significant populations of the tumor-initiating phenotypes described in the cancer stem cell literature. In this study, we observed that transplanting 293T cells into the cleared mammary fat pads of immunocompromised mice resulted in serially transplantable rapidly growing tumors. Propagation of spheres from tumor tissue or directly from the cell line enriched for ALDH activity and CD44+/CD24- markers and expression of EMT markers as well as genes that promote stem cell survival. Papers characterizing similar features in existing cancer cell lines have demonstrated a wide range in each of the purported cancer stem cell populations in these lines making the lines available to screen simultaneously against each surrogate limited.

The 293T cell line was developed through transformation of human embryonic kidney cells using the sheared Adenovirus 5 (Ad5) DNA [24]. It expresses genes permitting the replication of E1-deficient, non-replicating adeno and adeno-associated viral vectors leading to their common use of generating viral vectors for laboratory and gene therapy use. From the initial report in 1977, the transformation was surprisingly inefficient. Only two of eight transformations were successful with only one of twenty cultures forming a single colony in each case. Attempts to isolate these were not successful, however and a single transformed colony was observed in one dish not discarded 75 days after the original experiment. Partial serum starvation (2%) was used to select for the transformed phenotype and eventually the isolated culture was expanded. The culture was reported to contain cells of several morphologies of generally epitheliod appearing cells. Injection of 2 × 10^7 ^cells subcutaneously into nude mice yielded no tumors after 6-8 weeks. Re-injection at that time resulted in tumors 15-20 weeks after the first injection in 3/20 animals. In stark contrast, Shen et al [26] report that in cells passaged greater than 65 times, the tumorgenicity increases to 100% with tumors formed in two weeks. We have reproduced these findings with 100% tumor formation when injected into the cleared mammary fat pads of immunocompromised mice and further report that these tumors are serially transplantable with similarly aggressive biology and resembled poorly differentiated, high grade malignant primary tumor on histology. It's noteworthy that there is controversy on the origins of HEK 293T cells as it has been assumed that they were generated by transformation of either a fibroblastic, endothelial or epithelial cell all of which are abundant in kidney. However, the fact that the cells originated from cultured kidney cells does not say much about the exact cellular origin of the HEK 293T, as embryonic kidney cultures may contain small numbers of almost all cell types of the body. In fact, a study by Graham and colleagues provided evidence that these cells and several other human cell lines generated by adenovirus transformation of human embryonic kidney cells have many neuronal progenitor properties, suggesting that the adenovirus was taken up and transformed a neuronal lineage cell in the original kidney culture [41]. Thus, the spheres generated from 293T cells under stem-cell promoting culture condition may have features observed in neurospheres.

Given the in vivo and histological findings, we explored known cancer stem cell surrogates and signaling pathways described in various cancer studies and confirm this cell line faithfully recapitulates the described biology of purported cancer stem cells. Previous studies showed that nuclear and cytoplasmic extracts from undifferentiated cells can reprogram 293T cells into a stem cell-like phenotype with activation of embryonic stem cell genes and outgrowth of spherical structures. As a result of treatment of 293T monolayer cells with embryonic carcinoma cell extract, spherical colonies of 293T cells develop and induce Oct4 gene expression which in turn induces the expression several Oct4-responsive genes like Sox2, Nanog and Rex1 [42-44]. Consistent with these findings, our study has found activation of the pluripotency genes as well as formation of spheres in suspension culture suggesting that the stem cell promoting culture condition promotes dedifferentiation. The notion that cells with properties of cancer stem cells can be generated from differentiated somatic cells has been proposed in a recent study by Dean and colleagues [45]. These authors demonstrated that culturing retinoblastoma-deficient mouse embryonic fibroblasts in suspension culture form spherical structures, re-express embryonic stem cell markers and at least a subset of this population adopts characteristics of cancer stem cells. Likewise, recent accounts from Weinberg's group [46] and others [47] suggest the possibility of reversion or reprogramming of differentiated cancer cells into cancer stem cells.

Several studies from our lab have shown that breast cancer stem/progenitor cells are resistant to radiation compared to bulk, differentiated cells [9,16,48,49]. Phillips and colleagues also demonstrated the radioresistance of putative breast cancer stem/progenitor cells by comparing the radiosensitivity of cells derived from the CD44+/CD24-subpopulation of MCF-7 cell line grown as spheres vs. monolayers [8]. The resistance of cancer stem cells to radiation has been shown to be mediated by the β-catenin [9,49] or Notch signaling pathway [8]. Similar findings were reported in brain tumors demonstrating that CD133-expressing glioma stem cells are relatively resistant to radiation in vivo and in culture compared to non-stem cells [50]. Likewise, in our study the stem cell-enriched population from the 293T cells is much more resistant and with increased expression of β-catenin and Notch1 compared to the adherent population. Moreover, a study by Li et al demonstrated that the percentage of cancer stem cells, assayed by mammosphere forming efficiency, was significantly increased after chemotherapy highlighting the chemoresistant nature of cancer stem cells [7].

The molecular basis underlying the EMT process involves multiple changes in expression and function of proteins that include vimentin and cadherins [51-53]. Acquired expression of vimentin by carcinoma cells often symbolizes mesenchymal-like cell transformation [52] while loss of E-cadherin or gain of N-cadherin on tumor cell surface is frequently observed in malignant carcinomas and also correlated with enhanced aggressiveness and dedifferentiation [51]. Recent studies have demonstrated that induction of EMT using Twist and Snail in transformed mammary epithelial cells creates populations that are highly enriched for cancer stem cells as evidenced by increased CD44+/CD24- expression, mammosphere formation and tumor seeding ability [18]. These cells showed attributes of mesenchymal phenotype including expression of vimentin, fibronectin and n-cadherin. Similarly, in a recent study in pancreatic cell lines the knockdown Zeb1, another EMT-inducer, affected not only the initial formation of spheres but also led to reduction of sphere numbers in subsequent generations indicating its role in self-renewal and maintenance of the stem cell phenotype[13]. In our current study, we found higher expression of these EMT transcription factors in spheres from 293T cells vs. monolayer cells in line with the stem cell properties of 293T spheres.

MicroRNAs are emerging as important regulators of cellular differentiation and EMT. Recent findings have associated let-7 microRNAs with stem cell maintenance and loss of let-7 in cancer has been reported to cause dedifferentiation and self-renewal of breast cancer cells [37]. Consistent with our finding that the 293T spheres have markedly reduced Let-7 expression and increased H-ras and HMGA2, two known let-7 targets these authors showed that cancer stem cell population have markedly reduced let-7 and increased H-ras and HMGA2 expression. By expressing let-7 in cancer stem cells, the authors also found that let-7 regulates self renewal, multipotent differentiation, and the ability to form tumors [37]. Several recent studies have identified the miR-200 family and miR-205 as key regulators of EMT and enforcers of the epithelial phenotype of cancer cells [38,40]. MiR-200 was found to directly target the mRNA of the E-cadherin transcriptional repressors Zeb1 and Zeb2. While ectopic expression of miR-200 caused up-regulation of E-cadherin in cancer cell lines and reduced their motility, its inhibition reduced E-cadherin expression, increased expression of vimentin and induced EMT [40]. Another microRNA, miR-335, has been identified as metastasis suppressor microRNA in human breast cancer [39]. It inhibits metastatic cell invasion and migration through targeting of the progenitor cell transcription factor Sox4 and extracellular matrix component tenascin C. Moreover, the loss of expression of this microRNA is associated with poor distal metastasis-free survival [39].

## Conclusions

293T cells readily form spheres that are enriched with cancer stem cell surrogates ALDH1 and CD44+/CD24- and express the proteins associated with the epithelial-mesenchymal transition and stem cell developmental pathways. They are also depleted in the microRNA that has been implicated in cancer stem cell self-renewal. Of particular interest, these cells passage readily on serial transplant into the cleared mammary fat pad of immunocompromised mice. We propose that this is a valuable cell line to study the cancer stem cell surrogates identified in human breast cancer as well as the plasticity of transformed embryonic cells.

## Methods

### Cell culture

For sphere formation, 293T cells were placed in ultralow attachment dishes (Corning, NY) in mammosphere media as previously described by Dontu et al. [12]. Briefly, single cells were seeded in 6-well ultralow attachment plates (Corning, NY) in serum free MEM media supplemented with 20 ng/ml bFGF, 20 ng/ml EGF, and B27 (Invitrogen). Adherent 293T cells were grown on standard tissue culture plate in growth media that contains DMEM with 10% fetal bovine serum and penicillin-streptomycin.

### RT-PCR and miRNA analysis

Total RNA was isolated from cells 2D monolayer and 3D spheres with TRIzol reagent according to the manufacture's protocol. After treatment with DNase I (Ambion), two micrograms of the RNA samples were reverse-transcribed with random hexamers using Super Script III First-Strand Synthesis System from Invitrogen. Control reactions excluded reverse transcriptase. PCR was done with aliquots of the cDNA samples at the annealing temperature 60°C with the following primers: Oct4 forward 5'-ccagtatcgagaaccgagtgag-3', reverse 5'-gcatagtcgctgcttgatcg-3'; Nanog forward 5'-gatgcctcacacggagactg-3', reverse 5'-gctggggtaggtaggtgctg-3'; Sox2 forward 5'-tccacactcacgcaaaaacc-3', reverse 5'-ttgcaaacttcctgcaaagc-3'; Klf4 forward 5'-ttctcacctgtgtgggttcg-3', reverse 5'-cctgggtcttgaggaagtgc-3'; Snail forward 5'-cggaagcctaactacagcgag-3', reverse 5'-cttggcctcagagagctgg-3'; slug forward 5'-gatgcatattcggacccacac-3', reverse 5'-cctcatgtttgtgcaggagag-3'; Gapdh forward 5'-cacccagaagactgtggatgg-3', reverse 5'-ttctagacggcaggtcaggtc-3'. For microRNAs, small RNAs were extracted using a mirVana miRNA isolation kit (Ambion, Austin, TX) following the manufacture's protocol. Two micrograms of the total RNA sample was reverse-transcribed with RT miRNA First Strand Kit (SABiosciences, Frederick, MD). The cDNA was mixed with SYBR Green/ROX qPCR Master Mix and the mixture was added into a 96-well miRNA PCR Array (SABiosciences) that included primer pairs for 88 human miRNAs. PCR was performed on 7300 Real-Time PCR equipment (Applied Biosystems, Foster city, CA). Threshold cycle (Ct) for each miRNA was extracted using SDS2.3 software (Applied Biosystems) and data was analyzed using an Excel-based data analysis system (SA Bioscencies) that is based on the ΔΔCt method. Normalization of the raw data was made to one or more of the housekeeping genes in the Array.

### Immunoblotting

Cell extracts were prepared from 2D monolayer and 3D sphere cultures of 293T cells using cell lysis buffer (Cell Signaling, MA), supplemented with protease inhibitor (1 mM phenylmethanesulfonyl fluoride, PMSF). Protein concentrations were determined using the Bio-Rad Protein Assay (Bio-rad, CA) and 40 ug of each sample was separated using 4-20% Tris-HCl polyacrylamide gel (Invitrogen, CA) and transferred onto a PVDF membrane from Bio-Rad. The membranes were blocked in 5% milk powder in TBS-T for ~1 hour and were then incubated with the following primary antibodies: anti-E-cadherin, anti-N-cadherin, and anti-Vimentin (BD Biosciences); anti-zeb1 and anti-Rex1 (Abcam); anti-β catenin and anti-survivin (Cell Signal); and anti-notch 1 (Santa Cruz). Membranes were washed three times and incubated with the corresponding secondary antibody conjugated with a horseradish peroxidase in 5% nonfat milk at room temperature. After incubation with secondary antibody, the membranes were washed three times and immunoreactivity was detected by enhanced chemiluminescence. Anti-actin and anti-vincullin (Sigma) antibodies were used as loading controls.

### Flow Cytometry

For analyzing the CD44/CD24 subpopulation, 293T cells cultured under sphere and adherent conditions were harvested with 10 mM EDTA, centrifuged and resuspended in phosphate buffered saline (PBS) (10^5 ^cells/ml). They were then incubated with FITC-conjugated CD44 and PE-conjugated CD24 antibodies (BD Biosciences, San Diego, CA, USA) for 30 minutes at concentrations recommended by the manufacturer. Cells incubated in PBS, FITC or PE alone served as controls. Cell analysis for the expression of CD44 and CD24 was performed using a Beckman Coulter machine and the data files were analyzed using FlowJo software (Treestar, Ashland, OR). For aldehyde dehydrogenase (ALDH) activity assay, the Aldefluor kit (Lonza) was used according to the manufacturer's instructions. Briefly, about 5 × 10^5 ^2D monolayer and 3D sphere cultures of 293T cells were suspended in Aldefluor assay buffer containing ALDH substrate and incubated for 30 min at 37°C. As a negative control for each sample, a sample of cells was incubated with 50 mmol/L of the specific ALDH inhibitor diethylaminobenzaldehyde (DEAB). Aldefluor fluorescence was excited at 488 nm and fluorescence emission was detected using a Beckman Coulter machine and the data files were analyzed using FlowJo software (Treestar, Ashland, OR).

### Label retention assay

Cells were trypsinized, resuspendend in PBS, and labeled with PKH26 (Sigma). Labeled cells were plated in suspension in ultralow attachment plates in mammosphere culture media [12]. After 7 days, spheres were harvested, enzymatically dissociated and subjected to serial passages without further addition of PKH26. Intact spheres from the serial passages were subjected to PKH26 staining and were viewed by confocal microscopy.

### Clonogenic assay/3D sphere formation assay

This was conducted as we previously described [16]. Briefly, monolayer cultures of 293T cells were trypsinized into single cells and were seeded into individual wells of a 6-well tissue culture plate (for 2D) or ultralow attachment plates (for 3D). Both 2D and 3D 6-well plates containing seeded single cells were exposed to increasing doses of irradiation 4 hrs after plating. 2D plates were incubated for 14 days and colonies were stained with crystal violet while 3D cells were incubated in mammosphere media for 7 days, the spheres were stained with MTT and those with a size of 50 uM were counted using a Gelcount colony counter (Oxford Optronix, Oxford, UK). Survival curves were generated using Sigmaplot 8.0.

### Transplantation

2×10^6 ^fresh cells from monolayer culture were mixed with matrigel (1:1), and injected directly into the epithelium-free cleared fat pads of three-week-old female SCID/Beige recipient mice [54]. Mice were palpated weekly and tumor growth measured using calipers. When tumors reached 10 mm in diameter, they were collected for further analysis. Fresh tumor fragments were minced into 1 mm^3 ^fragments, and re-transplanted into new SCID/Beige hosts for subsequent transplant generations, and expanded as transplantable tumor tissue lines.

### Immunostaining procedure

Immunostaining was performed in a autostainer (Vision Biosystems, Norwell, MA) using Bond max protocol. The primary antibodies that were used in the study included E-cadherin (HECD-1, 1:100, Zymed/Invitrogen), β-catenin (1:500, BD Biosciences), VEGF (A-20; 1:10, Santa Cruz Biotechnology) and Keratin 19 (RCK 108, 1:50, Dako). The slides were dewaxed, rinsed with alcohol for dehydration and antigen retrieval was performed using citrate buffer. The slides were then treated with 3% H_2_O_2, and _primary antibodies were applied on the tissue sections and incubated for 10 minutes. The slides were subsequently treated with Poly-horseradish peroxidase, anti-mouse/rabbit IgGs. For visualization of the signal, 3, 3'diaminobenzidine (DAB) was used as the chromogen. Hematoxylin counterstaining was used for visualization of the nucleus.

## Competing interests

The authors declare that they have no competing interests.

## Authors' contributions

BGD, XZ, AAR, MDL, WX carried out experiments; collection and/or assembly of data by BGD, WX, AAR, MDL, HG, EC, LLi; data analysis and interpretation by BGD, LL, WX, SK, FR, JMR, AL, NTU, MTL, WAW; Financial support by WAW, MC, TAB, MTL; Manuscript writing by BGD, WAW; Final approval of manuscript by all authors.
